# Out-of-hospital cardiac arrest patients with an initial non-shockable rhythm could be candidates for extracorporeal cardiopulmonary resuscitation: a retrospective study

**DOI:** 10.1186/s13049-020-00800-2

**Published:** 2020-10-14

**Authors:** Atsunori Tanimoto, Kazuhiro Sugiyama, Maki Tanabe, Kanta Kitagawa, Ayumi Kawakami, Yuichi Hamabe

**Affiliations:** Tertiary Emergency Medical Center, Tokyo Metropolitan Bokutoh Hospital, 23-15 Kohtohbashi, 4-Chome, Sumida-ku, Tokyo, 130-8575 Japan

**Keywords:** Extracorporeal cardiopulmonary resuscitation, Non-shockable rhythm, Out-of-hospital cardiac arrest

## Abstract

**Background:**

Extracorporeal cardiopulmonary resuscitation (ECPR) is a promising treatment for refractory out-of-hospital cardiac arrest (OHCA). Most studies evaluating the effectiveness of ECPR include patients with an initial shockable rhythm. However, the effectiveness of ECPR for patients with an initial non-shockable rhythm remains unknown. This retrospective single-center study aimed to evaluate the effectiveness of ECPR for patients with an initial non-shockable rhythm, with reference to the outcomes of OHCA patients with an initial shockable rhythm.

**Methods:**

Adult OHCA patients treated with ECPR at our center during 2011–2018 were included in the study. Patients were classified into the initial shockable rhythm group and the non-shockable rhythm group. The primary outcome was the cerebral performance category (CPC) scale score at hospital discharge. A CPC score of 1 or 2 was defined as a good outcome.

**Results:**

In total, 186 patients were eligible. Among them, 124 had an initial shockable rhythm and 62 had an initial non-shockable rhythm. Among all patients, 158 (85%) were male, with a median age of 59 (interquartile range [IQR], 48–65) years, and the median low flow time was 41 (IQR, 33–48) min. Collapse was witnessed in 169 (91%) patients, and 36 (19%) achieved return of spontaneous circulation (ROSC) transiently. Proportion of female patients, presence of bystander cardiopulmonary resuscitation, and collapse after the arrival of emergency medical service personnel were significantly higher in the non-shockable rhythm group. The rate of good outcomes at hospital discharge was not significantly different between the shockable and non-shockable groups (19% vs. 16%, *p* = 0.69). Initial shockable rhythm was not significantly associated with good outcome after controlling for potential confounders (adjusted odds ratio 1.58, 95% confidence interval: 0.66–3.81, *p* = 0.31). In the non-shockable group, patients with good outcomes had a higher rate of transient ROSC, and pulmonary embolism was the leading etiology.

**Conclusions:**

The outcomes of patients with an initial non-shockable rhythm are comparable with those having an initial shockable rhythm. OHCA patients with an initial non-shockable rhythm could be candidates for ECPR, if they are presumed to have reversible etiology and potential for good neurological recovery.

## Background

Recently, extracorporeal cardiopulmonary resuscitation (ECPR) has been reported to be a promising treatment for refractory out-of-hospital cardiac arrest (OHCA) [1–3]. In OHCA patients with an initial shockable rhythm, several studies have reported better survival and neurological outcome in patients treated with ECPR compared with conventional cardiopulmonary resuscitation (CCPR) [4, 5]. However, there are only few reports evaluating the efficacy of ECPR in OHCA patients with an initial non-shockable rhythm. For in-hospital cardiac arrest patients treated with ECPR, non-shockable rhythm is more frequent as the initial rhythm than shockable rhythm [6, 7]. Even in OHCA patients, some case series have reported successful treatment with ECPR in selected etiologies such as pulmonary embolism and accidental hypothermia [8, 9]. We hypothesized that the selected OHCA patients with initial non-shockable rhythm could be candidate for ECPR. This study aimed to evaluate the efficacy of ECPR in OHCA patients with an initial non-shockable rhythm, with reference to the outcomes of patients with an initial shockable rhythm at our center.

## Methods

### Patients

This retrospective study included OHCA patients who were treated with ECPR at the tertiary emergency care center of Tokyo Metropolitan Bokutoh Hospital between January 2011 and December 2018. Patients aged below 18 years were excluded from the study. The patients were classified into two groups according to the initial rhythm: the shockable rhythm group and the non-shockable rhythm group. Data of the baseline demographic and clinical characteristics of the patients were obtained from patient’s medical records, and the timing of pre-hospital events was recorded according to the reports of emergency medical service (EMS) personnel. The institutional review board of Tokyo Metropolitan Bokutoh Hospital approved the study (institutional approval reference number 02–003), which complied with the tenets of the Declaration of Helsinki. The requirement for informed consent was waived owing to the retrospective design of the study.

### Protocol for ECPR and post-cardiac arrest care

In Japan, EMS personnel are required to transport every OHCA patient to a hospital performing cardiopulmonary resuscitation. They are permitted to perform defibrillation and advanced airway placement (e.g., use of tracheal intubation and supraglottic devices) and administer adrenaline, where indicated.

The indications for ECPR at our institution are as follows: (i) OHCA patients aged ≤65 years with an initial shockable rhythm and witness and (ii) OHCA patients aged ≤70 years with presumed reversible etiology who collapsed after the arrival of EMS personnel. In this case, any initial rhythm was acceptable, and the reversibility of the etiology was speculated based on the medical history and symptoms recorded by EMS personnel. Patients with very long transfer times and terminal illnesses were excluded. The implementation of ECPR was decided at the discretion of the individual emergency physician. Therefore, some cases did not meet the rigid indications.

ECPR was implemented immediately after the patients’ arrival at the emergency room. In all cases, we selected the ipsilateral or contralateral femoral vein and artery for insertion of venous and arterial cannulas. Further, 16-French (Fr) and 22-Fr cannulas were chosen for femoral artery and vein, respectively. Cannulation was performed percutaneously using the Seldinger technique under ultrasonic guidance. When our emergency room was renovated in August 2014, an interventional radiology-computed tomography (CT) system was installed [10]. Before this (until July 2014), a cannula was placed under ultrasonic guidance only, and after August 2014, cannulas were placed under both ultrasonic and fluoroscopic guidance. The extracorporeal membrane oxygenation (ECMO) circuit, consisting of a centrifugal pump, a hollow fiber oxygenator (MERA CPB circuit; Senko Medical Instrument Mfg. Co. Ltd., Tokyo, Japan, or Capiox EBS; Terumo Corporation, Tokyo, Japan), and a heparin-coated surface circuit, was primed using normal saline with 3000 units of heparin. After the successful insertion of the venous and arterial cannulas, the ECMO circuit was connected and the ECMO pump flow was set at 4 L/min initially. After the initiation of ECMO, a 4-Fr sheath was placed in the superficial femoral artery to prevent distal limb ischemia. The conventional cardiopulmonary resuscitation was performed parallel to the cannulation until efficient ECMO pump flow was achieved.

After the initiation of ECMO, all patients were resuscitated according to the recommendations at each period [11–14]. They received appropriate amounts of fluid or vasopressors to maintain the mean blood pressure above 65 mmHg and were placed under ventilation to maintain normocarbia and adequate oxygenation. Patients in whom cardiac etiology was suspected underwent coronary angiography (CAG) and percutaneous coronary intervention, if indicated. Patients who remained comatose after ECMO initiation were treated with targeted temperature management at 34 °C for 24 h and subsequently rewarmed to 36 °C for the next 12 h using a heat exchanger in the circuit.

Neurological outcomes were predicted according to the results of clinical examinations performed at least 72 h after the return of spontaneous circulation (ROSC) and of brain CT. Neurological outcomes were predicted as poor when (1) a patient remained unconscious for at least 72 h post-ROSC with a Glasgow Coma Scale motor response score of ≤2; (2) there was no pupillary reflex; and (3) diffuse anoxic injury was recognized post-ROSC or during follow-up brain CT (on days 4–5) [12, 14]. Even in these patients, we did not withdraw treatment, and ongoing life-sustaining measures were retained. However, additional aggressive treatment modalities such as hemodialysis and additional mechanical circulatory support devices were withheld.

### Outcomes

The cerebral performance category (CPC) scale was used to assess the outcomes of neurological function at hospital discharge [15], and this was the primary outcome. The CPC scale ranges from 1 to 5 with 1 representing intact function and 5 representing brain death. The outcome of neurological function was considered good if the CPC score was 1 or 2, and poor if the CPC score was 3–5. The CPC scores were determined by reviewing the report from the rehabilitation department.

### Statistical analysis

Continuous variables were reported as medians with interquartile ranges (IQRs), and dichotomous variables were reported as numbers with percentages. Univariate analysis was performed using Fisher’s exact test for categorical variables and the Mann-Whitney U test for continuous variables. All reported *p*-values were two-tailed, and values less than 0.05 were considered to indicate statistical significance.

First, the rate of good neurological outcome was compared between the shockable and non-shockable rhythm groups. A multivariate logistic regression analysis was then performed to evaluate the association between the initial rhythm and good neurological outcomes to control for potential confounders. We selected potential confounders that appeared to be clinically important by referring to those used in previous studies (age, bystander CPR, witness, low flow time, and transient ROSC) [16].

Second, the background characteristics of the patients were compared between those with good and poor neurological outcomes in the non-shockable group.

All statistical analyses were performed using EZR (Saitama Medical Center, Jichi Medical University, Saitama, Japan) [17], which is a graphical user interface for R (The R Foundation for Statistical Computing, Vienna, Austria).

## Results

**Fig. 1:**
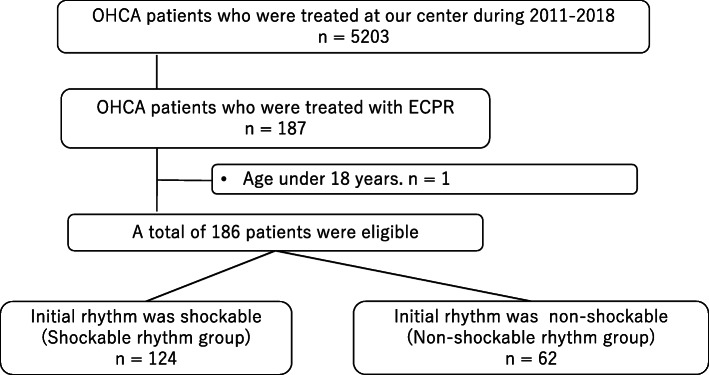
Flow chart of patient selection. OHCA, out-of-hospital cardiac arrest

A total of 5203 OHCA patients were transferred to our center during the study period. Of them, 187 were treated with ECPR for refractory cardiac arrest. One patient aged below 18 years was excluded, and a total of 186 patients were eligible for the study. Among them, 124 patients had an initial shockable rhythm and 62 patients had an initial non-shockable rhythm (Fig. 1).

The background characteristics of all patients and patients in each group are shown in Table 1. Of the total 186 patients, 158 (85%) were male, with a median age of 59 (IQR, 48–65) years. Collapse was witnessed in 169 (91%) patients, and 119 (64%) patients received bystander CPR. Fifty-five (30%) patients collapsed after the arrival of EMS personnel. Median low flow time was 41 (IQR 33–48) min. Thirty-six patients (19%) achieved ROSC transiently and collapsed again during the period between the collapse and the initiation of ECMO.

**Table 1:** Characteristics of all patients and each group

	All patients	Non-shockable rhythm	Shockable rhythm	p-value
	*n* = 186	*n* = 62	*n* = 124	
Age, median (IQR)	59 (48, 65)	60 (49, 66)	58 (46, 65)	0.24
Sex, male, n (%)	158 (85)	46 (74)	112 (90)	< 0.01
Witness, n (%)	169 (91)	59 (95)	110 (89)	0.19
Bystander CPR, n (%)	119 (64)	51 (82)	68 (55)	< 0.01
Cardiac arrest after EMS personnel arrival, n (%)	55 (30)	41 (66)	14 (11)	< 0.01
Low flow time, median (IQR)	41 (33, 48)	40 (31, 51)	41 (34, 48)	0.84
Transient ROSC, n (%)	36 (19)	12 (19)	24 (19)	1
Etiology, n (%)				0.06
Acute coronary syndrome	77 (41)	22 (36)	55 (44)	
Vasospastic angina	5 (3)	2 (3)	3 (2)	
Idiopathic ventricular fibrillation	42 (23)	1 (2)	41 (33)	
Cardiomyopathy	16 (9)	4 (6)	12 (10)	
Pulmonary embolism	10 (5)	9 (15)	1 (1)	
Accidental hypothermia	3 (2)	2 (3)	1 (1)	
Aortic dissection	11 (6)	6 (10)	5 (4)	
Respiratory failure	7 (4)	7 (11)	0	
Others	15 (8)	9 (15)	6 (5)	
Coronary angiography	137 (74)	31 (50)	106 (86)	< 0.01

*CPR* cardiopulmonary resuscitation, *EMS* emergency medical service, *IQR* interquartile range, *ROSC* return of spontaneous circulation

The proportion of female patients, presence of bystander CPR, and collapse after the arrival of EMS personnel were significantly higher in the non-shockable rhythm group. Low flow time was not significantly different. In the shockable rhythm group, the most frequent etiology of cardiac arrest was acute coronary syndrome (ACS) (55 patients), followed by idiopathic ventricular fibrillation (41 patients) and cardiomyopathy (12 patients). In the non-shockable rhythm group, the most frequent etiology was also ACS (22 patients), followed by pulmonary embolism (nine patients), and respiratory failure (seven patients). CAG was performed more frequently in the shockable rhythm group than in the non-shockable rhythm group.

Among all patients, 55 (30%) survived to hospital discharge, and 34 (18%) had good neurological outcomes at hospital discharge. The rate of good neurological outcomes at hospital discharge was not significantly different between the shockable and non-shockable rhythm groups (19% vs. 16%, *p* = 0.69). Additionally, the rate of survival at hospital discharge was not different between the two groups (33% vs. 23%, *p* = 0.17) (Table 2).

**Table 2:** Outcomes of all patients and each group

	All patients	Non-shockable rhythm	Shockable rhythm	p-value
	n = 186	n = 62	n = 124	
Survival, n (%)	55 (30)	14 (23)	41 (33)	0.17
Good neurological outcome, n (%)	34 (18)	10 (16)	24 (19)	0.69
Successful weaning from ECMO, n (%)	86 (46)	26 (42)	60 (48)	0.44

*ECMO* extracorporeal membrane oxygenation

After controlling for potential confounders, we found that the adjusted odds ratio of the initial shockable rhythm as compared to that of the non-shockable rhythm for good neurological outcomes was 1.58 (95% confidence interval: 0.66–3.81, *p* = 0.31). In this model, the presence of bystander CPR and transient ROSC was significantly associated with good neurological outcomes (Table 3).

**Table 3:** Multivariate logistic regression analysis of the prognostic factors of good neurological outcomes

	Adjusted odds ratio	95% confidence interval	p-value
Initial shockable rhythm	1.58	0.66–3.81	0.31
Age	0.99	0.97–1.03	0.97
Bystander CPR	3.6	1.30–9.95	0.01
Low flow time	0.97	0.94–1.01	0.1
Witness	1.19	0.23–6.23	0.84
Transient ROSC	5.04	2.12–12.00	< 0.01

*CPR* cardiopulmonary resuscitation, *ROSC* return of spontaneous resuscitation

In the non-shockable group, patients with good neurological outcomes were predominantly female, with a higher rate of transient ROSC than that in those with poor neurological outcomes. Pulmonary embolism was the most frequent etiology in patients with good neurological outcomes (Table 4).

**Table 4:** Background characteristics of the patients with good and poor neurological outcomes in the non-shockable group

	Good neurological outcome	Poor neurological outcome	p -value
	*n* = 10	*n* = 52	
Age, median (IQR)	62 (54, 65)	59 (49, 66)	0.66
Sex, male, n (%)	4 (40)	42 (81)	0.01
Low flow time, median (IQR)	33 (26, 45)	42 (32, 52)	0.24
Transient ROSC, n (%)	5 (50)	7 (14)	0.02
Initial rhythm, n (%)			1
PEA	9 (90)	44 (85)	
Asystole	1 (10)	8 (15)	
Etiology, n (%)			0.06
Acute coronary syndrome	2 (20)	20 (39)	
Vasospastic angina	1 (10)	1 (2)	
Idiopathic ventricular fibrillation	0	1 (2)	
Cardiomyopathy	1 (10)	3 (6)	
Pulmonary embolism	4 (40)	5 (10)	
Accidental hypothermia	1 (10)	1 (2)	
Aortic dissection	0	6 (12)	
Respiratory failure	1 (10)	6 (12)	
Others	0	9 (17)	

*IQR* interquartile range, *ROSC* return of spontaneous resuscitation

## Discussion

In the present study, the outcomes of patients with an initial non-shockable rhythm were compared with those of patients with an initial shockable rhythm among all patients treated with ECPR at our center. We found that the initial rhythm was not significantly associated with neurological outcome, and the neurological outcome of the non-shockable group was almost comparable with that of the shockable rhythm group. In the non-shockable rhythm group, pulmonary embolism was the leading etiology in patients with good neurological outcome, and the rate of transient ROSC during resuscitation was higher in patients with good neurological outcomes.

In the last decade, several studies have reported the efficacy of ECPR in patients with refractory OHCA. Most of these studies included patients with an initial rhythm of ventricular fibrillation or ventricular tachycardia [1, 2, 4, 18]. In another study evaluating the factors associated with good outcomes, the initial shockable rhythm has been reported to be a significant predictor of good outcome [19]. In patients with refractory cardiac arrest, the reversibility of both cardiac and brain function is the major determinant of the prognosis. OHCA patients with an initial shockable rhythm are more likely to have reversible etiology such as ACS. The leading cause of refractory ventricular fibrillation is reported to be ACS in most studies [4, 18]. Furthermore, sudden onset of lethal arrhythmia is commonly noted; therefore, normal cerebral perfusion and oxygenation were generally maintained prior to the event. This is considered an important factor contributing to good prognosis in patients with an initial shockable rhythm. Generally, OHCA patients with non-shockable rhythm had poor outcomes compared with those having shockable rhythm [20]. They tend to have various etiologies refractory to treatment, often experiencing the symptomatic period preceding the cardiac arrest, and they might suffer from shock or hypoxia before collapsing. These factors lead to the difficulty in treating the underlying etiology and achieving brain recovery when performing ECPR in patients with an initial non-shockable rhythm. However, several case reports have reported the efficacy of ECPR in OHCA patients with non-shockable rhythm. Patients with accidental hypothermia or pulmonary embolism have been reported to be treated successfully in these case reports [8, 9, 21–23]. Bunya et al. reported two cases of refractory cardiac arrest with an initial non-shockable rhythm and agonal respiration at the presentation. Both patients were treated with ECPR and had good neurological outcomes [24]. The results of the present study and the case series show that the initial non-shockable rhythm alone might not fully dismiss the possibility of ECPR in OHCA patients. If the patients had a reversible etiology and potential for good brain recovery, then the indication of ECPR even in those with non-shockable rhythm should be considered.

In the non-shockable rhythm group, patients with good neurological outcome had a higher rate of transient ROSC. The transient ROSC between the collapse and the initiation of ECMO might shorten the actual low flow time and lessen anoxic brain injury. Furthermore, pulmonary embolism is the leading etiology among patients with good outcome. Pulmonary embolism causes acute right heart failure, and veno-arterial ECMO could be a highly effective treatment for this condition [25, 26]. The results of our study emphasize the importance of appropriate etiologic diagnosis and the prediction of the potential for good brain recovery during cardiac arrest.

It is very challenging to diagnose the etiology of OHCA during cardiopulmonary resuscitation (CPR). The efficacy of point-of-care ultrasonography has been emphasized in patients during cardiac arrest. Cardiac tamponade, pulmonary embolism, and ACS could be diagnosed with this method [27]. However, the evaluation of potential for brain function recovery during CPR is further challenging. There are some promising physiological parameters such as presence of agonal breathing [28], regional cerebral oxygen saturation [29], and quantitative pupillometry [30] that have been reported to be associated with the neurological outcome and might indicate the residual function of the brain. These diagnostic methods and neurological physiological parameters during CPR should be investigated in future studies to establish the appropriate indication criteria for ECPR beyond the initial rhythm and to improve the effectiveness of ECPR.

There are several important limitations to this study. First, this was a retrospective single-center study with a small sample size, and the statistical power was limited. Second, the indication of ECPR at our center for patients with non-shockable rhythm is relatively limited, which includes only patients who collapse after the arrival of EMS personnel and are speculated to have treatable etiology estimated from the symptom before collapse. This protocol aimed to shorten the low flow time and to obtain a more precise patient history at the scene. However, one-third of patients actually collapsed before the arrival of EMS personnel because the final decision was at the discretion of the attending physician. As a result, there was no significant difference in the low flow time between patients with shockable rhythm and those with non-shockable rhythm. The results of this study emphasize the importance of patient selection but do not demonstrate the efficacy of ECPR in unselected patients with an initial non-shockable rhythm. Third, neurological outcomes were evaluated only at the time of hospital discharge, and long-term outcomes were not evaluated. We evaluated the CPC score as the scale of neurological outcome, and this was rated according to the report of the rehabilitation department. Interestingly, this score has shown interrater variability [31]; however, as it was not evaluated in this study, this may be considered as a limitation. Fourth, the outcome of ECPR in patients with OHCA is influenced by the system of emergent medical services and pre-hospital treatment. Therefore, the results of this study could not be generally applied to various clinical settings. Finally, the cost-effectiveness of the method was not evaluated in this study. Some studies reported that ECPR could be cost-effective, particularly in patients with an initial shockable rhythm [32–34]. The cost-effectiveness of ECPR in patients with non-shockable rhythm should be evaluated in future studies.

## Conclusions

Among the OHCA patients treated with ECPR at our center, the outcomes of patients with an initial non-shockable rhythm were comparable to those of patients with an initial shockable rhythm. OHCA patients with an initial non-shockable rhythm could be considered as candidates for ECPR, if they are presumed to have reversible etiology and the potential for good neurological recovery. Further studies focusing on the diagnosis and prognostication during cardiac arrest are needed.

## Data Availability

The datasets used and/or analyzed during the current study are available from the corresponding author on reasonable request.

## Abbreviations

ACS: Acute coronary syndrome
CAG: Coronary angiography
CCPR: Conventional cardiopulmonary resuscitation
CPC: Cerebral performance category
CPR: Cardiopulmonary resuscitation
CT: Computed tomography
ECPR: Extracorporeal cardiopulmonary resuscitation
ECMO: Extracorporeal membrane oxygenation
EMS: Emergency medical service
IQRs: Interquartile range
OHCA: Out-of-hospital cardiac arrest
ROSC: Return of spontaneous circulation

## References

[1] Maekawa K, Tanno K, Hase M, Mori K, Asai Y (2013). Extracorporeal cardiopulmonary resuscitation for patients with out-of-hospital cardiac arrest of cardiac origin. Crit Care Med.

[2] Stub D, Bernard S, Pellegrino V, Smith K, Walker T, Sheldrake J (2015). Refractory cardiac arrest treated with mechanical CPR, hypothermia, ECMO and early reperfusion (the CHEER trial). Resuscitation..

[3] Beyea MM, Tillmann BW, Iansavichene AE, Randhawa VK, Van Aarsen K, Nagpal AD (2018). Neurologic outcomes after extracorporeal membrane oxygenation assisted CPR for resuscitation of out-of-hospital cardiac arrest patients: a systematic review. Resuscitation..

[4] Sakamoto T, Morimura N, Nagao K, Asai Y, Yokota H, Nara S (2014). Extracorporeal cardiopulmonary resuscitation versus conventional cardiopulmonary resuscitation in adults with out-of-hospital cardiac arrest: a prospective observational study. Resuscitation..

[5] Bartos JA, Carlson K, Carlson C, Raveendran G, John R, Aufderheide TP (2018). Surviving refractory out-of-hospital ventricular fibrillation cardiac arrest: critical care and extracorporeal membrane oxygenation management. Resuscitation..

[6] D’Arrigo S, Cacciola S, Dennis M, Jung C, Kagawa E, Antonelli M (2017). Predictors of favourable outcome after in-hospital cardiac arrest treated with extracorporeal cardiopulmonary resuscitation: a systematic review and meta-analysis. Resuscitation..

[7] Andersen LW, Holmberg MJ, Løfgren B, Kirkegaard H, Granfeldt A (2019). Adult in-hospital cardiac arrest in Denmark. Resuscitation..

[8] Mandigers L, Scholten E, Rietdijk WJR, den Uil CA, van Thiel RJ, Rigter S (2019). Survival and neurological outcome with extracorporeal cardiopulmonary resuscitation for refractory cardiac arrest caused by massive pulmonary embolism: a two center observational study. Resuscitation..

[9] Sawamoto K, Bird SB, Katayama Y, Maekawa K, Uemura S, Tanno K (2014). Outcome from severe accidental hypothermia with cardiac arrest resuscitated with extracorporeal cardiopulmonary resuscitation. Am J Emerg Med.

[10] (JA-HERS) T founding members of the JA for HERS (2019). The hybrid emergency room system: a novel trauma evaluation and care system created in Japan. Acute Med Surg.

[11] Neumar RW, Otto CW, Link MS, Kronick SL, Shuster M, Callaway CW (2010). Part 8: adult advanced cardiovascular life support: 2010 American Heart Association guidelines for cardiopulmonary resuscitation and emergency cardiovascular care. Circulation..

[12] Deakin CD, Nolan JP, Soar J, Sunde K, Koster RW, Smith GB (2010). European resuscitation council guidelines for resuscitation 2010 section 4. Adult advanced life support. Resuscitation..

[13] Callaway CW, Donnino MW, Fink EL, Geocadin RG, Golan E, Kern KB (2015). Part 8: post–cardiac arrest care. Circulation..

[14] Nolan JP, Soar J, Cariou A, Cronberg T, Moulaert VR, Deakin CD (2015). European resuscitation council and European Society of Intensive Care Medicine Guidelines for post-resuscitation care 2015: section 5 of the European resuscitation council guidelines for resuscitation 2015. Resuscitation..

[15] Jennett B, Bond M (1975). Assessment of outcome after severe brain damage. Lancet..

[16] Lee SW, Han KS, Park JS, Lee JS, Kim SJ (2017). Prognostic indicators of survival and survival prediction model following extracorporeal cardiopulmonary resuscitation in patients with sudden refractory cardiac arrest. Ann Intensive Care.

[17] Kanda Y (2013). Investigation of the freely available easy-to-use software “EZR” for medical statistics. Bone Marrow Transplant.

[18] Yannopoulos D, Bartos JA, Raveendran G, Conterato M, Frascone RJ, Trembley A (2017). Coronary artery disease in patients with out-of-hospital refractory ventricular fibrillation cardiac arrest. J Am Coll Cardiol.

[19] Debaty G, Babaz V, Durand M, Gaide-Chevronnay L, Fournel E, Blancher M (2017). Prognostic factors for extracorporeal cardiopulmonary resuscitation recipients following out-of-hospital refractory cardiac arrest. A systematic review and meta-analysis. Resuscitation..

[20] Sasson C, Rogers MAM, Dahl J, Kellermann AL (2010). Predictors of survival from out-of-hospital cardiac arrest a systematic review and meta-analysis. Circ Cardiovasc Qual Outcomes.

[21] Huang H, Chiu C, Yen H, Chen Y, Siao F (2015). Prolonged pulseless electrical activity : successful resuscitation using extracorporeal membrane oxygenation. Am J Emerg Med.

[22] Kuhnke M, Albrecht R, Schefold JC, Paal P (2019). Successful resuscitation from prolonged hypothermic cardiac arrest without extracorporeal life support: a case report. J Med Case Rep.

[23] Miyazaki K, Hikone M, Kuwahara Y, Ishida T, Sugiyama K, Hamabe Y (2019). Extracorporeal cardiopulmonary resuscitation for massive pulmonary embolism in a “hybrid emergency room”. Am J Emerg Med.

[24] Bunya N, Wada K, Yamaoka A, Kakizaki R, Katayama Y, Kasai T (2019). The prognostic value of agonal respiration in refractory cardiac arrest: a case series of non-shockable cardiac arrest successfully resuscitated through extracorporeal cardiopulmonary resuscitation. Acute Med Surg.

[25] George B, Parazino M, Omar HR, Davis G, Guglin M, Gurley J (2018). A retrospective comparison of survivors and non-survivors of massive pulmonary embolism receiving veno-arterial extracorporeal membrane oxygenation support. Resuscitation..

[26] Kjaergaard B, Kristensen JH, Sindby JE, de Neergaard S, Rasmussen BS (2019). Extracorporeal membrane oxygenation in life-threatening massive pulmonary embolism. Perfusion..

[27] Long B, Alerhand S, Maliel K, Koyfman A (2018). Echocardiography in cardiac arrest: an emergency medicine review. Am J Emerg Med.

[28] Debaty G, Labarere J, Frascone RJ, Wayne MA, Swor RA, Mahoney BD (2017). Long-term prognostic value of gasping during out-of-hospital cardiac arrest. J Am Coll Cardiol.

[29] Nishiyama K, Ito N, Orita T, Hayashida K, Arimoto H, Beppu S (2015). Regional cerebral oxygen saturation monitoring for predicting interventional outcomes in patients following out-of-hospital cardiac arrest of presumed cardiac cause: a prospective, observational, multicentre study. Resuscitation..

[30] Behrends M, Niemann CU, Larson MD (2012). Infrared pupillometry to detect the light reflex during cardiopulmonary resuscitation: a case series. Resuscitation..

[31] Grossestreuer AV, Abella BS, Sheak KR, Cinousis MJ, Perman SM, Leary M (2016). Inter-rater reliability of post-arrest cerebral performance category (CPC) scores. Resuscitation..

[32] Dennis M, Zmudzki F, Burns B, Scott S, Gattas D, Reynolds C (2019). Cost effectiveness and quality of life analysis of extracorporeal cardiopulmonary resuscitation (ECPR) for refractory cardiac arrest. Resuscitation..

[33] Kawashima T, Uehara H, Miyagi N, Shimajiri M, Nakamura K, Chinen T (2019). Impact of first documented rhythm on cost-effectiveness of extracorporeal cardiopulmonary resuscitation. Resuscitation..

[34] Bharmal MI, Venturini JM, Chua RFM, Sharp WW, Beiser DG, Tabit CE (2019). Cost-utility of extracorporeal cardiopulmonary resuscitation in patients with cardiac arrest. Resuscitation..

